# X-Ray Structure of the Human Calreticulin Globular Domain Reveals a Peptide-Binding Area and Suggests a Multi-Molecular Mechanism

**DOI:** 10.1371/journal.pone.0017886

**Published:** 2011-03-15

**Authors:** Anne Chouquet, Helena Païdassi, Wai Li Ling, Philippe Frachet, Gunnar Houen, Gérard J. Arlaud, Christine Gaboriaud

**Affiliations:** 1 Institut de Biologie Structurale Jean-Pierre Ebel, CEA, Grenoble, France; 2 Institut de Biologie Structurale Jean-Pierre Ebel, UJF Grenoble 1, Grenoble, France; 3 Department of Clinical Biochemistry and Immunology, Statens Serum Institut, Copenhagen, Denmark; 4 Institut de Biologie Structurale Jean-Pierre Ebel, CNRS, Grenoble, France; University of Queensland, Australia

## Abstract

In the endoplasmic reticulum, calreticulin acts as a chaperone and a Ca^2+^-signalling protein. At the cell surface, it mediates numerous important biological effects. The crystal structure of the human calreticulin globular domain was solved at 1.55 Å resolution. Interactions of the flexible N-terminal extension with the edge of the lectin site are consistently observed, revealing a hitherto unidentified peptide-binding site. A calreticulin molecular zipper, observed in all crystal lattices, could further extend this site by creating a binding cavity lined by hydrophobic residues. These data thus provide a first structural insight into the lectin-independent binding properties of calreticulin and suggest new working hypotheses, including that of a multi-molecular mechanism.

## Introduction

Calreticulin (CRT) is an intriguing multi-compartmental protein involved in many cellular processes with broad patho-physiological implications [1], [2]. In addition to its well-characterized function as a carbohydrate and non-native-peptide-recognizing chaperone and Ca^2+^-signaling molecule in the endoplasmic reticulum [3], CRT was shown to play a key role in the MHC class I assembly pathway [4], [5]. CRT also moves to the cell surface and extracellular milieu, where it is involved in a variety of other important functions, such as cell adhesion, migration and proliferation, or wound healing [1], [2]. Finally CRT was recently proposed to be a surface ‘eat-me’ signal of dying cells, also conserved in drosophila [6]–[8]. The molecular and structural determinants involved in these various CRT properties still need to be deciphered, except for the lectin binding site [9].

**Figure 1:**
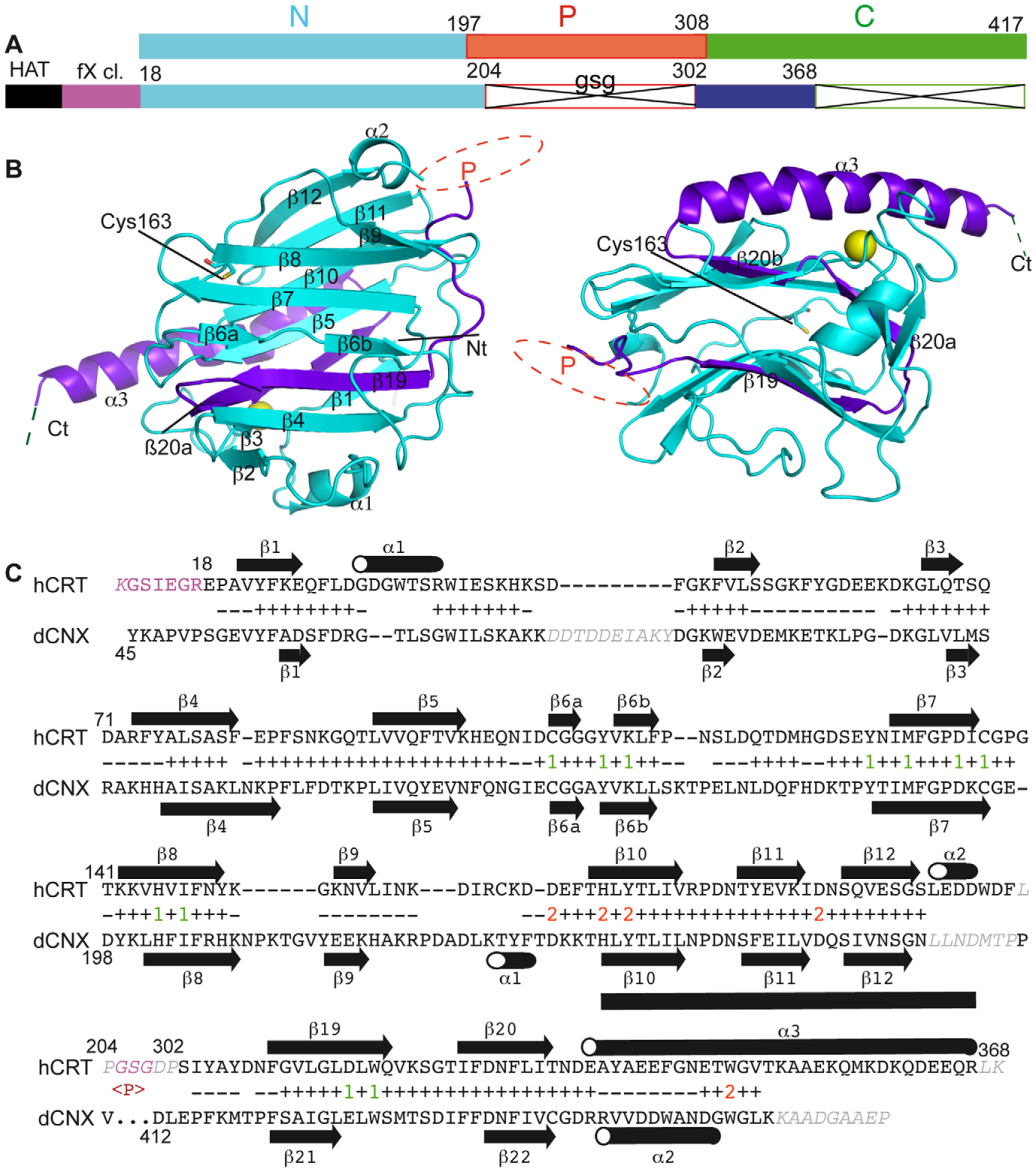
Structure of the human CRT globular domain. (A) Representation of the linear structure of CRT and of the construct used in this study. N, P, C, the discontinuous CRT segments as defined usually. Amino acid numbering is that of the unprocessed polypeptide. HAT, His-tag; fX cl., factor X cleavage site. (B) Two different views of the CRT globular domain structure. Regions from the N and C segments are colored light blue and dark blue, respectively. The Ca^2+^ ion is represented as a golden sphere. The approximate location of the P-domain insertion is indicated. (C) Structural alignment of human CRT and dog CNX. The positions of ß-strands and α-helices are indicated. The N-terminal extension and the linker are shown in pink. Grey italics show residues not defined in the structure. Residues aligned (−) or defining the common core of human CRT and rat CNX (+) are indicated. Residues included in the conserved clusters 1 and 2 are labeled 1 (green) and 2 (red), respectively.

CRT and its membrane-bound homologue calnexin (CNX) both comprise an extended proline-rich P-domain inserted between N- and C-terminal domains, called N and C, the latter bearing a highly charged extremity (Figure 1). The pioneering crystallographic analysis of a large fragment from CNX has revealed an extended P-domain inserted into a globular legume lectin-like domain [10], but available structural data for CRT have long been restricted to the NMR structure of its P-domain [11]. To gain further structural insights on this protein, we have solved the X-ray structure of the human CRT globular domain. The structure is compared to that of CNX [10] and of the lectin domain of mouse CRT released very recently [9]. Two patches of evolutionary conserved surface residues are described, emphasizing their atypical properties and functional roles. Careful analysis of the crystal packing interactions reveals that the disordered N-terminal extension binds to the edge of the lectin site and that an intriguing CRT molecular zipper could provide a larger binding platform. These observations provide a new structural basis for the non-lectin chaperone activity of CRT.

## Results and Discussion

### Designing a CRT fragment suitable for X-ray crystallography

Due to their flexible nature, the P-domain and the C-terminal extension of CRT were expected to preclude crystallization of the full-length protein. We therefore designed a truncated protein in which the P-domain was replaced by a GSG linker and the C-terminal residues 369–417 were deleted (Figure 1A). The P-domain boundaries were deduced from the X-ray structure of CNX and the NMR structure of the rat CRT P-domain [10], [11]. These domain limits (residues 204–302) slightly differ from those currently found in sequence databases (residues 197–308) (Figure 1A). The size of the C-terminal deletion was more difficult to predict. Several constructs were produced, and the fragment ending at Lys368 gave the best results in terms of crystallization. A similar construct, with the same C-terminal end, was used to solve the X-ray structure of the mouse CRT lectin domain [9].

In keeping with previous observations [12], monomeric CRT coexisted with higher molecular weight forms after the affinity purification step, and these were removed by gel filtration chromatography (see Methods). Mass spectrometry analysis of the purified material yielded a value of 32,201 Da (predicted mass: 32,202 Da). The thermal stability of the recombinant domain was determined using a thermofluor assay [13], yielding a relatively low Tm value (40°C), consistent with other analyses [9], [14]. As this domain contains a Ca^2+^-binding site, calcium was used throughout the purification process. Crystallization of this fragment proved to be successful (see Methods), allowing crystallographic analyses.

### High-resolution structure of the human CRT globular domain

The structure of the human CRT globular domain (Figure 1B) was solved at a resolution of 1.55 Å and refined to *R*_work_ and *R*_free_ values of 0.176 and 0.190, respectively (Table 1). At such a resolution, most of the residues are very clearly defined. Only residues 203–204 and 302–303, on either side of the linker, and the C-terminal Leu-Lys residues were not clearly seen in the electron density. The two crystal forms obtained at different pH (8.5–9.0 and 5.7–7.0) yielded similar structures, except for their N-terminal extension.

**Table 1:** Data collection and refinement statistics.

	1^st^ crystal form	2^nd^ crystal form
**PDB code**	3POS	3POW
**Data collection**		
ESRF Beamline	Id29	Id23-eh2
Space group	P2_1_2_1_2_1_	P2_1_2_1_2_1_
Cell (a, b, c) Å	42.2, 91.1, 194.0	42.9, 70.4, 91.8
Resolution range high/low (Å)	1.65–20	1.55–10.0
Last resolution shell high/low (Å)	1.65–1.7	1.55–1.59
Observed reflections a	1416077 (20909)	187458 (14576)
Unique reflections^a^	88382 (6434)	39833 (3013)
Redundancy^a^	16 (3.2)	4.7 (4.8)
Completeness (%)^a^	96.8 (83.0)	96.8 (99.9)
I/SigI^a^	18.8 (2.4)	15.2 (3.0)
Rsym (%)^a^	11.7 (45.4)	7.0 (53.5)
**Refinement**		
Resolution range high/low Å	1.65–19.9	1.55–10.0
Last resolution shell high/low (Å)	1.65–1.69	1.55–1.59
R_work_/R_free_ (%)^a^^,^ b	17.0/20.7 (21.2/22.2)	17.6/19.0 (24.4/26.2)
R.m.s.d. Bound/Angle (Å/°)	0.01/1.07	0.009/1.16
Mean B factor (Å^2^)	19.3	16.8

a Values in parentheses are for outermost shell.

b 5% of the structure factors were isolated to monitor R_free_.

Like its counterparts in CNX and mouse CRT, the human CRT globular domain exhibits a jelly-roll fold assembled from two anti-parallel ß-sheets, one convex and one concave. Remarkably, the C domain inserts into the globular domain, providing the central strands ß19 and ß20 of the ß-sandwich structure (Figure 1B). The long C-terminal α3 helix is kinked at Thr346. Subtle differences in this kink orientation induce a 1.5 Å displacement at the end of α3 between different molecules of the first crystal form. Interestingly, the free Cys163, mutated to Ser in the mouse CRT globular domain, is not accessible in this conformation, as it is buried between residues from strands ß7 and ß10. Otherwise, this structure is quite similar to that of mouse CRT [9], with a r.m.s. deviation of only 0.4 Å based on 242 superposed Cα positions. Likewise, the Ca^2+^ ion is also coordinated by 7 ligands contributed by the backbone carbonyls of Gln26, Lys62 and Lys64, two water molecules, and both side-chain carboxyl oxygens of Asp328.

The human CRT and dog CNX globular domains exhibit an r.m.s. deviation of 1.6 Å based on 213 superimposed Cα positions, this value dropping to 0.77 Å when considering a common core defined by 142 Cα positions (see Figure 1C). Despite this overall homology, these two structures differ in certain regions (Figures 1B, 1C). CRT lacks the N-terminal extension present in CNX, and thus its N- and C-terminal ends lie on opposite faces of the structure. Compared to CNX, the C-terminal helix of CRT is prolonged by a large extension (residues 347–366). Two other α-helical structures, α1 (residues 29–35) and α2 (residues 196–199) are present in CRT. The loop before strand ß2, and those between ß8 and ß9 are much shorter in CRT. Strand ß9 and the following loop are slightly different in the two structures. Helix α1 observed in CNX is absent in human CRT.

### Two surface patches are common to CRT and CNX

**Figure 2:**
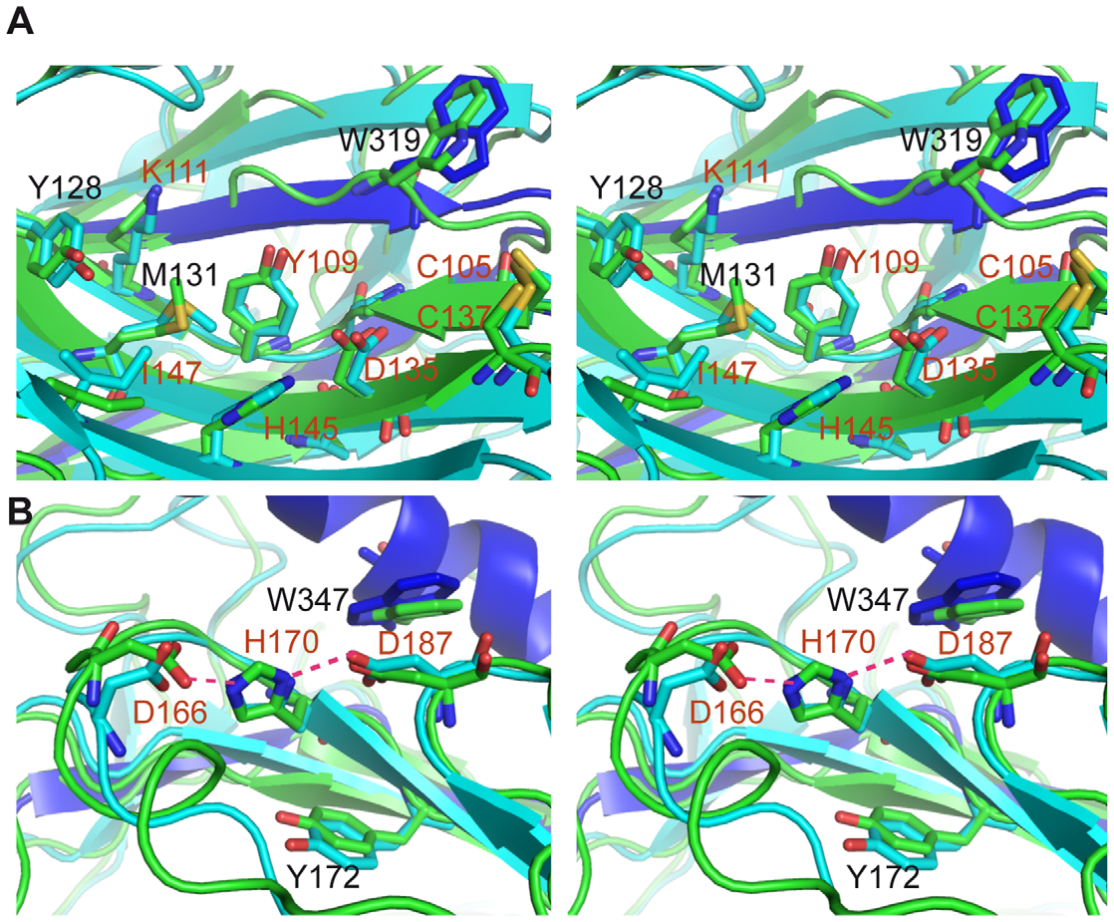
Surface patches common to CRT and CNX. (A) The more extended patch, cluster 1, corresponding to the identified lectin site [9]. (B) The smaller cluster 2. CRT and CNX structures are in light blue and green, respectively. Labels correspond to CRT residues, and red labels indicate the most conserved ones. Hydrogen bonds are represented by dotted red lines. The two patches are displayed using stereograms.

Although CRT and CNX have common folds, most of their surface-exposed side chains are different, with the striking exception of two patches of residues. The most extended patch is located on the concave ß-sheet surface (Figure 2A). From a functional standpoint, this first cluster corresponds to the lectin site of CRT and CNX, as identified by mutagenesis experiments and recently deciphered by the X-ray structure of the mouse CRT lectin domain complexed to a tetrasaccharide [9]. The most conserved residues in this patch are Cys105 and Cys137 engaged in a disulfide bridge and the nearby Gly107; Tyr109, Pro134 and Asp135 in the middle of the patch; Lys111 and the neighboring residues His145 and Ile147 on the edge. Less conserved residues Trp319, Met131 and Tyr128 also contribute to the patch. This cluster includes many charged residues that are mostly buried: Lys111 (>75% buried), Asp135 (>60%), Asp317 (about 80%). The central Tyr109 is also buried (80%), except for its hydroxyl group that is always protonated at physiological pH because of its high pKa value (>12).

A second smaller patch of conserved surface residues comprises Asp166 (233 in CNX), His170 (237), Tyr172 (239), Asp187 (254) and Trp347 (456) (Figure 2B). These side-chains are superimposed in CRT and CNX despite significant differences in the conformation of the preceding loop (Figure 2B). In the central Asp/His/Asp triad, the His residue is wedged in-between the two charged Asp side chains at H-bonding distances (Figure 2B). This atypical environment had not been described so far. The strong sequence conservation of these residues (Figures S1, S2) further suggests that they may have functional implications. In accord with this hypothesis, the His170Ala mutation was shown to dramatically impair the CRT chaperone function *in vivo* [15]. It was initially proposed that this effect could be due to a conformational change that would extend up to the lectin site [15]. However, this site is about 20 Å away from His170, on the opposite side of the beta-barrel (Figure S3). How this mutation could alter the lectin site at such a distance is therefore unclear. The hypothesis of a large conformational change was based in part on the observation that the His170Ala mutation induces changes in Trp fluorescence and circular dichroism [15]. However, the circular dichroism spectra of the wild-type and mutant proteins had similar shapes, and the mutant was produced *in vivo* at the same level as the wild-type species [15]. In the X-ray structure, Trp347 lies in close proximity to Asp187 (3.2 Å, Figure 2B). The His170Ala mutation is therefore expected to modify the stability and charge of Asp187, altering in turn the spectral properties of the neighboring Trp347. Therefore, even a local destabilization of the DHD cluster is likely to alter the Trp fluorescence and circular dichroism spectrum of CRT. Thus, although this question warrants further experimental investigation, we believe that this second patch of residues could also support a functional role, in light of its striking structural and sequence conservation and in agreement with previous *in vivo* data.

### A peptide-binding site on the edge of the lectin site

**Figure 3:**
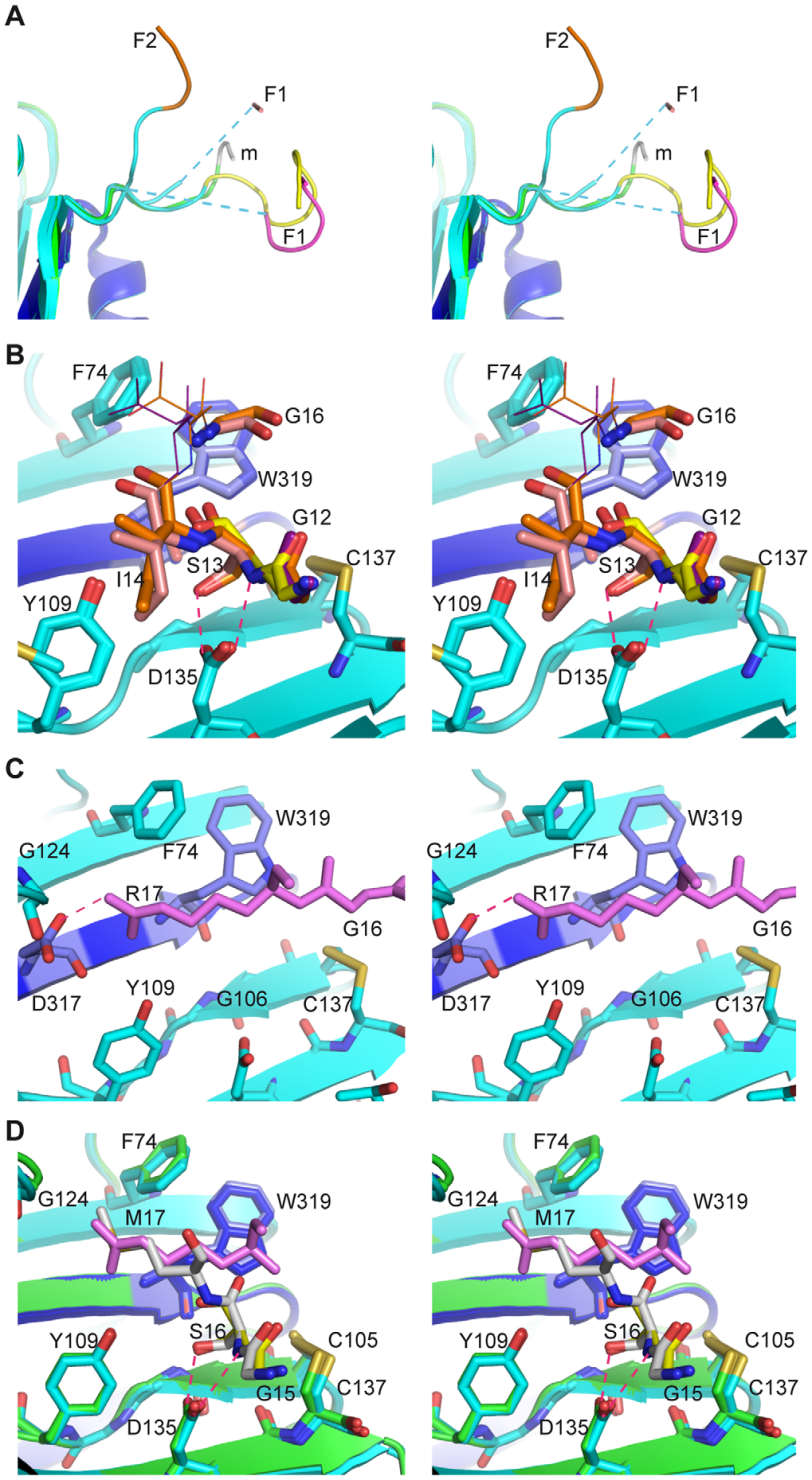
The N-terminal extension of CRT interacts with the edge of the lectin site. (A) Superposed views of the N-terminal extensions. (B) The interaction mode observed in the first crystal form. (C) The interaction mode observed in the second crystal form. (D) Superposition of the interacting regions of human CRT and the corresponding region of mouse CRT. Labels correspond to interacting residues of human CRT in (B) and (C), and to mouse CRT in (D). Hydrogen bonds and ionic interactions are represented by dotted red lines. The blue dotted lines in (A) represent disordered parts. F1, F2: crystal forms 1 and 2; m: mouse CRT. All items are displayed as stereograms.

CNX and CRT also act as chaperones on non-glycosylated substrates [3], [16] and the CNX globular domain has been shown to bind peptides through a hitherto unidentified site that has been proposed to be distinct from the lectin site [17]. In this respect, our crystallographic analyses of the human CRT globular domain reveal interesting polypeptide-binding properties. The unfolded N-terminal extension fused to CRT for purification purposes is indeed consistently found to interact with the edge of the lectin site, and the quality of the electron density shows that this interaction is specific. To achieve such an interaction, this extension adopts a wide range of conformations, with some disordered parts, emphasizing its flexible nature (Figure 3A). No crystal was obtained when the preceding HAT tag was present. No interaction was observed in the crystal packing between this edge of the lectin site and properly folded segments of the CRT molecules. Two different modes of interaction were observed. The first mode involves H-bonding between Asp135 and the hydroxyl group and peptide bond nitrogen of Ser13. Three such interactions were observed in the first crystal form (Figure 3B). A distinct interaction mode, involving an ionic bond between Asp317 and Arg17, was observed in the second crystal form (Figure 3C). Interestingly, inspection of the carbohydrate-free crystal form (pdb code 3O0V) obtained by Kozlov *et al* (2010) also reveals an interaction between the N-terminal Gly-Ser-Met extension and the edge of the lectin site. Moreover, Ser16 and Met17 of the mouse CRT construct are strikingly superposed to the corresponding residues Ser13 and Arg17 of our human CRT construct, as observed in the first and second crystal forms, respectively (Figure 3D). Altogether, these independent observations convincingly map a peptide-binding site comprising Phe74, Trp319, Cys105, Cys137, and Asp135 (Figures 3 and S3). This site mainly contributes hydrophobic contacts, except for the polar interactions described above. Phe46, at the bottom of the Met17 binding pocket, also contributes to the hydrophobic environment. Additional contacts provided by Met131, Asp317 (Figure 3B), Gly106 (Figure 3C), or Gly124 (Figure 3D) are also observed but seem secondary to the main interaction site. Thus, we may have identified two sub-sites of the hitherto unknown peptide-binding site of CRT. This site overlaps the carbohydrate-binding site of the homologous mouse CRT lectin domain recently identified by X-ray analysis [9], suggesting that different types of ligands can bind to the edge of the lectin site. This would explain why Trp319, involved in all the peptide-binding interactions depicted here, was shown to be essential to the CRT *in vivo* chaperone activity and to its *in vitro* ability to prevent thermal aggregation of MDH, a non glycosylated substrate [18]. The same effect was also true, to a lesser extent, for the Cys105–Cys137 disulfide bond [18], which is also at the edge of this common carbohydrate/peptide-binding area. On the other hand, several mutations have been shown to suppress the lectin activity of CRT or CNX without affecting other carbohydrate-independent functions, such as suppression of the aggregation of thermally denatured non-glycosylated proteins [17], [19], *in vitro* peptide-binding [17], and chaperoning of class I histocompatibility molecules [20]. These mutations map a particular area of the lectin domain, the one that specifically recognizes the glucose unit (Figure S3). The corresponding residues include Y109, K111, Y128 and D317 of CRT [19], [20], as well as Y166, M169, I184 of CNX, which are homologous to Y128, M131 and I147 in CRT [17]. Interestingly, these residues are located on the side opposite to the peptide-binding site described above (Figure S3), and their mutations into alanine will not affect the peptide-binding property. Thus, even though the proposed peptide-binding site lies on the edge of the lectin site, in an area where carbohydrate-binding also occurs, this location is fully compatible with previous mutagenesis data.

### An intriguing CRT molecular zipper

Most of the residues in the proposed peptide-binding site are common to the CRT and CNX families, but Phe74 is specific to CRT. It is conserved in most of the CRT sequences, except for a group of plant CRTs (Figure S4). Intriguingly, the preceding CRT-specific 69–73 SQDAR segment largely contributes to a crystal packing interaction omnipresent in the crystal forms obtained so far. Ionic bonds between Asp71 and Arg162, and between Arg73 and Glu167 are central to this interaction. These are complemented by hydrophobic contacts between Val50 and Pro139, and by H-bonding interactions bridging the main-chain atoms of Ser69 and Asp71 to the tip of Lys142 and Asp165 side-chains, respectively. These mainly polar interactions thus represent the most favorable CRT/CRT interface, at least in the crystalline state, since they have been observed three times in the first crystal form, and once in the second form. We have also checked that these interactions occur consistently in the crystal packing of the mouse CRT lectin domain (pdb codes 3O0V, 3O0W, 3O0X), thus providing four additional observations. Finally, these interactions are observed over a wide range of pH (5.5–9.0), whereas the interactions mediated by the nearby His42 and His123 change polarity in this pH range, thus giving rise to two different crystal forms in our study.

**Figure 4:**
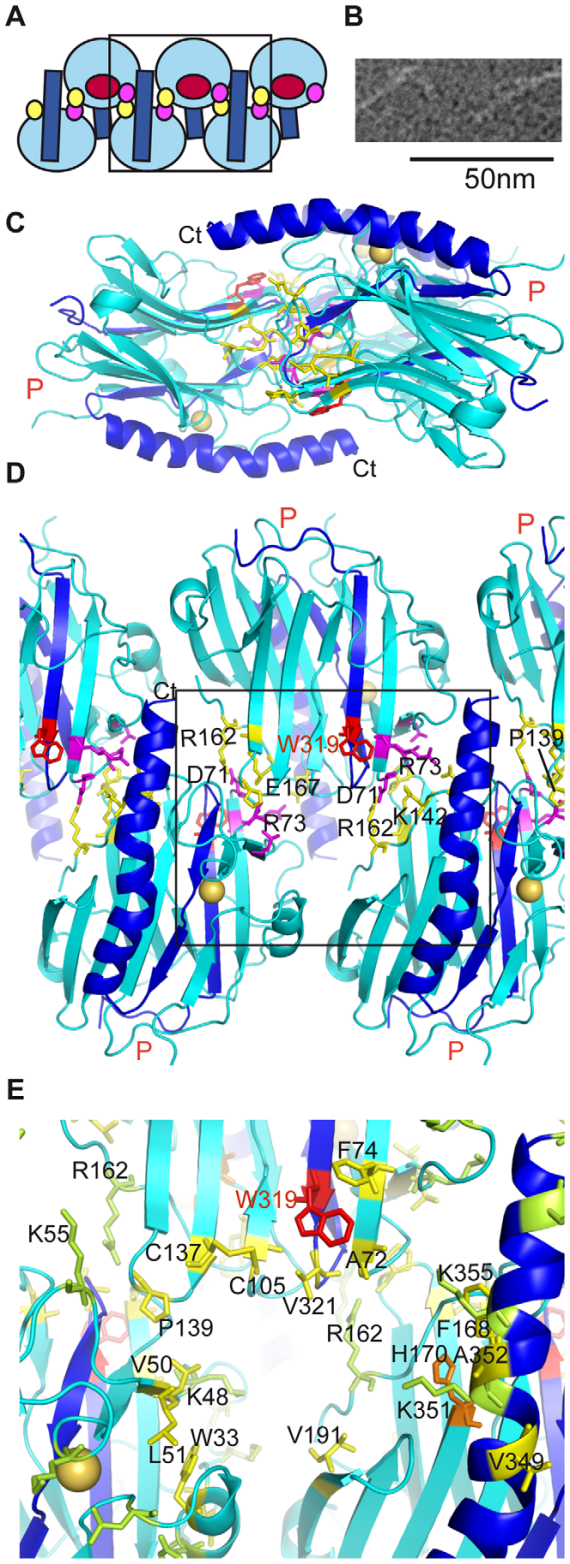
The CRT molecular zipper. (A) Schematic representation of the zipper. A red circle indicates the position of the binding site. Small circles locate the relative position of the SQDAR segment (pink) and its interacting residues in the neighboring molecule (yellow). The C-terminal helices are drawn in dark blue. (B) Negative-staining electron micrograph of the CRT construct. (C, D) Two orthogonal side- and top-views (the latter framed in A) of the interactions between CRT molecules defining the molecular zipper as seen in the crystal lattice. Residues involved in hydrogen and ionic bonds are shown in pink (segment SQDAR) and yellow. P marks the insertion position of the missing P domain. (E) Detailed view of the pocket lining the binding site as framed in (D). Exposed hydrophobic residues lining the pocket are in yellow; charged residues exposing a large hydrophobic surface (>65 Å^2^), forming a secondary shell, are in pale green; Residues shown by mutation to be essential for the chaperone function of CRT are in red (W319) and orange (H170).

Further analysis of this set of conserved interactions reveals intriguing properties. First, a molecular zipper is formed when these interactions are applied on both sides of the CRT domain (residues 71–73 and 162–167) (Figure 4A). It is composed of two lines of molecules with inverted orientations, one with the ligand-binding site up, the other down (Figure 4A), the long C-terminal helices providing both a flat basis and a flap (Figure 4C). Linear assemblies of CRT molecules are also seen on electron micrographs (Figure 4B). A close inspection of the area of the peptide-binding site reveals that it is next to a cavity formed by the 3 neighboring molecules (Figure 4D). Moreover, Trp319 comes across this cavity in relative proximity (10 Å) to the Asp-His-Asp triad of the neighboring molecule (Figure 4E), despite the fact that these elements lie on opposite sides of the same CRT molecule (Figure S3). Given that both His170 and Trp319 were shown to be essential for the *in vivo* chaperone activity of CRT [15], [18], this raises the intriguing question of a possible functional relevance of such a proximity. In addition, fully or partially exposed hydrophobic side-chains line the cavity in this area (Figure 4E), which further suggests that this may complement the above-described site for the binding of unfolded peptides. The cavity may complement as well the lectin site, introducing binding properties specific to CRT and distinct from those of CNX. Finally, it should be stressed that this set of interactions can likely be also achieved in full-length CRT, the C-terminal extension and the P-domain being located on two lateral sides of the array of molecules (Figures 4C, 4D).

### Conclusion

This high-resolution structure of the globular domain of human CRT provides a very accurate model for future investigations. Similar domain limits are observed for the human and mouse CRT globular domains [9], which both include the 18–202 segment from the N domain and the 303–366 segment from the C domain. Consistent structural domain limits must be used when dissecting CRT to locate functional units and binding sites. Moreover, several independent serendipitous observations lead to the first description of unfolded protein segments bound to CRT, on the outer edge of the lectin site. Further investigations of the binding specificity of this area might be possible by specifically designing the sequence of the N-terminal extension of CRT. On the edge of this peptide-binding site, the SQDAR segment, which is specifically found in CRT sequences, exhibits a strong propensity for interaction, at least with other CRT molecules. This raises the possibility for CRT to generate larger peptide-binding platforms. The molecular zipper formed in the crystals through the SQDAR-mediated interactions looks biologically relevant in several ways, especially if one considers that CRT is often associated to membrane surfaces, a situation that possibly stabilizes such elongated, thin and flat oligomers. As such, these observations provide a structural basis to reconsider in part the chaperone and other CRT functions as multi-molecular processes.

## Materials and Methods

### Protein expression and purification

All expression plasmids were derived from plasmid pHFX-CRT, containing the cDNA encoding full-length human CRT (MRC Gene Service, Cambridge, UK) fused at its N-terminus to an HAT tag followed by a factor X cleavage site. The P-domain (residues 204–302) was replaced by a GSG linker, as described elsewhere [21], and truncation of the C-terminal segment after residue 368 was obtained using the primer 5′-GGACAAACAGGACGAGGAGCAGAGGCTTAAGTAGTAAGCTTGCGGCCGCACTCGAGC-3′ and reverse. Transformed Rosetta 2(DE3) cells (Novagen) were optimally grown at 37°C and protein production was induced by 0.1 mM IPTG for 16–20 h at 20°C. Cells were lysed in 20 mM Tris-HCl, 0.3 M NaCl, 5 mM CaCl_2_, 10 mM imidazole, pH 7.5, using the FAST Prep matrix (MP Biomedicals). Purification was performed in the same buffer by affinity chromatography on a Ni^2+^-Sepharose resin (HiTrap™, Amersham). Fractions containing the CRT domain were further purified on a S75 16/60 prep grade gel filtration column (GE/Amersham). Fractions containing pure monomeric CRT, as checked by non-denaturing SDS-PAGE, were concentrated to 12 mg/mL. Control of the sample thermostability was performed at the EMBL htxlab facility using thermal-shift assays.

### Crystallization

Initial crystallization conditions were determined using the EMBL htxlab facility and commercial screens (QIAGEN), allowing generation of needle-like crystals. Optimal conditions were reproduced manually using the vapor diffusion method, at 4°C. Crystal form I was obtained using a reservoir solution containing 30% polyethylene glycol (PEG) 4000, 0.1–0.2 M sodium acetate, 0.1 M Tris-HCl, pH 8.5 or 9.0. Crystal form II was obtained using 30% PEG 4000, 0.2 M ammonium acetate, 10 mM magnesium acetate, and either 0.05 M MES, pH 5.7 or 6.0, or 0.05 M HEPES, pH 7.0.

### X-ray structure determination

The initial best diffracting crystal had a long needle-like shape (>250 µm) that allowed us to translate it to record several data sets at different exposure times. Data processing and scaling were performed using XDS [22]. The three most coherent data sets could be merged to obtain a highly accurate and redundant unique data set at 1.7 Å resolution. Data collection statistics are listed in Table 1. In this first crystal form, the space group is P2_1_2_1_2_1_, with cell dimensions a = 42.4 Å, b = 91.5 Å, c = 194.8 Å. The solvent content is about 40%, and the asymmetric unit contains 3 molecules. The structure was solved by iterative molecular replacements. The core structure comprising the main secondary structure elements of the CNX lectin domain [10] was used as a starting search model. After careful inspection of the electron density, a smaller search model was defined, restricted to the 130 best fitting residues. The correct molecular replacement solution was obtained using this second search model on the CaspR website (www.igs.cnrs-mrs.fr/Caspr2/index). Although the initial R-factor was high after the AMORE step [23], it dropped to 0.45 after 200 minimization steps using CNS refinement [24] at 3 Å resolution. That this solution was valid was confirmed by the observation of several residue substitutions in the electron density map, convergence of the CNS refinement minimizations steps using non-crystallographic restraints at 1.6 Å resolution, and improved iterative automatic reconstruction of this model using the ARP/wARP procedure [25]. Iterative reconstruction of the model was carried out using O [26], CNS, and the ARP/wARP procedure until recovery of most of the residues. Standard iterative rounds of manual building and refinement steps were finally carried out using BUSTER and Coot [27], [28].

Crystal form II diffracted to 1.55 Å. The space group is P2_1_2_1_2_1_, with cell dimensions a = 42.9 Å, b = 70.4 Å, c = 91.8 Å. The solvent content is about 45%, with a single molecule per asymmetric unit. The structure was easily solved using the Phaser molecular replacement software [29], using the structure of one molecule of crystal form I as a search model.

### Structure analysis

Two clusters of conserved surface residues were initially identified by visual inspection of the superimposed CNX and CRT structures. Comparison of the crystal contacts in the two crystal forms obtained in this study also revealed a CRT segment that is involved in the same crystal packing interactions.

The sequence conservation of the residues belonging to these particular clusters and segment was then carefully analyzed. For this purpose, several redundant tools have been used to analyze the degree of sequence conservation in the CRT/CNX family: CONSURF [30] and several other multiple sequence alignments generated on the PipeAlign website ([31], http://igbmc.u-strasbg.fr/PipeAlign) were used. Buried acidic and basic groups have long been known to be more particularly present at functionally important sites. Detection of such buried charged residues and prediction of their pKa value was performed using the ProPKA web server (http://propka.ki.ku.dk) [32].

## Supporting Information

Figure S1 **Sequence alignments of surface residues of cluster 2.** This sequence alignment shows how the residues displayed in Figure 2B (highlighted in yellow) are strongly conserved in the CRT/CNX sequence family. More detailed information about the sequence names is provided in Figure S2.
(PDF)

Figure S2 **Description of the set of sequences of the CRT/CNX family.** The following information about the sequence names is indicated: main protein name (when defined), source and alignment score of the whole sequence to the human globular domain sequence. The sequences have been automatically assigned to several groups using the pipealign procedure [30]. In the alignments, the sequence names directly coming from the Swissprot database have the SW prefix; the other protein sequences, translated from the gene, have the SPT prefix.
(PDF)

Figure S3 **Relative locations of the glucose-binding site, the peptide-binding site, cluster 2 and the main mutations affecting the** ***in vivo*** **chaperone properties.** A. Zoom on the lectin site. B. The lectin site and cluster 2 are located on two opposite sides of the beta-barrel. Mutations affecting only the lectin activity, which line the glucose-binding site (GBS) are colored magenta in A and green in B. The proposed peptide-binding site is colored orange in A, red and yellow in B. The red and orange residues in B highlight the mutations affecting the *in vivo* chaperone properties.
(PDF)

Figure S4 **Sequence alignments of the SQDAR 69–73 segment.** This sequence alignment shows how the residues highlighted in yellow are well conserved in the CRT groups 1 and 2; less conserved in groups 3 and 4; and not conserved in the group 5. The following F74 (green) is found in most of the CRT-like sequences, except for the plant group 4. It is not present in the CNX-like group 5 sequences. Details corresponding to the sequence names are given in Figure S2.
(PDF)
