# Supplementary material for: X-Ray Structure of the Human Calreticulin Globular Domain Reveals a Peptide-Binding Area and Suggests a Multi-Molecular Mechanism
Source: PLoS One. 2011 Mar 15;6(3):e17886. doi: 10.1371/journal.pone.0017886 (PMC3057994; doi:10.1371/journal.pone.0017886)
Supplement: Figure S1 — Sequence alignments of surface residues of cluster 2. This sequence alignment shows how the residues displayed in Figure 2B (highlighted in yellow) are strongly conserved in the CRT/CNX sequence family. More detailed information about the sequence names is provided in Figure S2. (PDF) [file pone.0017886.s001.pdf]

Figure S1: Sequence alignments of surface residues of cluster 2.
This sequence alignment shows how the residues displayed in Figure 2B (highlighted in yellow) are strongly conserved in the CRT /CNX sequence family. More detailed information about the sequence names is provided in Figure S2.

```
GROUP_1
human_GB_CRT          DEFTHLYTLIVRPDNTYEVKIDNS
SPT_A8E4J9_9ACAR      DVFTHLYTLIVKPDNTYVVKIDNE
SPT_Q64K99_9ACAR      DVFTHLYTLIVKPDNTYVVKIDNE
SPT_B5X0V5_SALSA      DELTHLYTLILNPDQTYEVKINNE
SW_CALR_RABIT         DEFTHLYTLIVRPDNTYEVKIDNS
SPT_Q64K76_IXOPE      DVYTHLYTLIVKPDNTYTVKIDNE
SPT_Q7ZXY3_XENLA      DSFTHLYTLIVRPDNTYEVKIDNS
SPT_Q68HD1_IXOSC      DVYTHLYTLIVKPDNTYTVKIDNE
SPT_B5X207_SALSA      DEYTHLYTLILNPDNTYEVKIDNK
SPT_Q64K83_9ACAR      DVYTHLYTLVLKPDNTYTVKIDNE
SW_CALR_MACFA         DEFTHLYTLIVRPDNTYEVKIDNS
SPT_Q9U6S0_STRPU      DEFTHLYTLIVKSDNSYEVRIDNE
SPT_A5D7J6_BOVIN      DEFTHLYTLIVRPNNTYEVKIDNS
SPT_A1YB06_EISFO      DTLSHLYTLIVRPDNTYEVKIDNK
SPT_Q98984_RANRU      DVYSHLYTLIVRPDNTYEVKIDNS
SPT_B3S3X6_TRIAD      DELTHLYTLILKSDNTYEVKIDNE
SPT_Q6NVT6_XENTR      DSFTHLYTLIVRPDNTYEVKIDNS
SPT_A5LGG9_CRAGI      DVFTHLYTLIVNPDNSYVVKIDNE
SW_CALR_CRIGR         DEFTHLYTLIVRPDNTYEVKIDNS
SPT_A6YIE3_PINFU      DVFTHLYTLIVNSDNTYEVRIDNE
SPT_Q6WSQ2_RHISA      DVFTHLYTLIVKPDNTYQIKIDNE
SPT_Q64K91_DERAN      DVFTHLYPLIVKPDNTYQVKIDNE
SPT_Q64K92_9ACAR      DVFTHLYTLIVKPDNTYQVKIDNE
SPT_Q0VJ74_9BILA      DELTHLYTLILRPDNTYEVQIDGE
SPT_Q64K89_DERVA      DVFTHLYTLIVKPDNTYQVKIDNE
SPT_O97372_DIRIM      DVFTHLYTLIVNSDNTYEVQIDGE
SPT_Q9PUC1_DANRE      DELTHLYTLILRPDQTYEVKIDNE
SPT_C5MRN9_9BILA      DELTHLYTLILNADNTYEVQIDGE
SPT_Q26268_APLCA      DVFSHLYTLIVRPDNTYEVKIDNE
SW_CALR_ONCVO         DVFTHLYTLIVNSDNTYEVQIDGE
SPT_A9C3S5_DANRE      DEYSHLYTLIVNPDNTYEVKIDNK
SPT_B8K275_FENCH      DVFSHLYTLIVNPDNTYEVLIDNE
SPT_Q8IS63_COTRU      DVYTHLYTLIVKPDNTYEVLIDNE
SPT_Q8WR36_ANOGA      DVFTHFYTLVVRADNTYEVLIDNE
SPT_Q17MI1_AEDAE      DVYTHFYTLVVKSDNTYEVLIDNE
SPT_C1BVC1_9MAXI      DAFTHLYTLILNTDRTYQVLIDNA
SPT_D3TM69_GLOMM      DVYTHFYTLIVKPDNTYEVLIDNE

GROUP_2
SPT_C6TCW1_SOYBN      DRLTHFYTFILRPDATYSVLVDNR
SPT_B6TA28_MAIZE      DKLTHVYTFILRPDASYSLLVDNR
SPT_C5XET1_SORBI      DKLTHFYTFILRPDASYSLLVDNR
SPT_B8LRK0_PICSI      DKLTHVYTLIIRPDASYSILIDNR
SPT_B4FUA8_MAIZE      DKLTHVYTFILRPDASYSLLVDNR
SPT_C5Z0S1_SORBI      DKLTHFYTFILRPDATYSILIDNR
SPT_Q8GUI1_ARATH      DKLNHFYTFILRPDASYSVLVDNK
SPT_B9MY68_POPTR      DKLTHFYTFILRPDASYSVLVDNR
SPT_Q8LJ85_ORYSJ      DKLTHVYTFILRPDASYSILVDNR
SPT_B8AZS4_ORYSI      DKLTHFYTFILRPDASYSLLVDNR

GROUP_3
SPT_Q4R3W0_MACFA      DGFTHLYTLILRPDLSYDVKIDGQ
SPT_B1Q2L7_MOUSE      DGFTHLYTLILRPDLSYEVKVDGQ
SW_CALR3_HUMAN        DGFTHLYTLILRPDLSYDVKIDGQ
SPT_Q3TQS0_MOUSE      NKDTHLYTLILRPNATYEVKIDNQ
SPT_D3ZYM6_RAT        NKDTHLYTLILRPNATYEVKIDNE
SPT_C1N041_9CHLO      DELTHVYTLRLHANNTYEVLIDNE
SPT_O45034_SCHJA      DQKTHLYTLIVRPDNSYEVLVDNE
SPT_C4Q5I7_SCHMA      DLKTHLYTLIVNPNNKYEVLVDNE
SPT_C8XTL8_9EUKA      DQLTHVYTLVVKPDNTYKIKIDGE
SPT_D3BIF1_POLPA      DQLSHQYTLILKPDNTYAVLIDNV
SW_CALR_EUGGR         DTLSHTYTAVIKADRTYEVLVDQV
SPT_C4M296_ENTHI      DDISHLYTLIIRPNNTYVVKIDGV
SPT_B7GDH4_PHATR      DSVTHLYTLVVRPDNTFEVFVDNK
SPT_Q967S4_TRYCO      DKLTHAYTLHIAPDNSYKFYLDGS
SPT_A4HJP8_LEIBR      EKATHVYTVQISPNNTYQLYVDGK
SPT_Q4Q601_LEIMA      DTATHVYTVEIAPNNTYQLYVDGM
SPT_Q4CPZ0_TRYCR      DKLTHVYTLHIAADNSYEFFLDGE
SPT_C8CIJ1_9TRYP      DKLTHVYTLRISSDDSYELYVDEE
SPT_Q9XYF8_TRYCR      DKLTHVYTLHIAADNSYEFFLDGE
SPT_C5L9W8_9ALVE      DTLSHVYRLVLRPNDTVRVEIDGE
SPT_C5LE92_9ALVE      DTSSHVYRLVLRPNDTVRVEVDGE
SPT_C5LZP6_9ALVE      DTLSHVYRLVLRPNDTVRVEIDGD
SPT_D0NR25_PHYIN      DTDAHLYSFVLKPDDTYEVKIDGK
SPT_A8PEL1_BRUMA      DELSHVYTLILYPNNSIQIFADYE

GROUP_4
SPT_Q7Y140_ORYSJ      DQLTHVYTLIIRPDAKYSILIDNT
SPT_A9SCB3_PHYPA      DKLSHVYTFIIRPDATYSILVDNK
SW_CALR_BERST         DQLTHVYTFILRPDASYSILIDNV
SPT_C4PB36_CARPA      DQLTHVYTFILRPDATYSILVDNV
SW_CALR1_ARATH        DQLTHVYTFVLRPDATYSILIDNV
SPT_A9NS22_PICSI      DQLTHVYTFILHPDATYSILIDNK
SPT_Q5MCL9_WHEAT      DQLSHVYTLIIRPDATYSILIDNE
SPT_O22502_BRANA      DQLTHVYTFILRPDATYTILVDNV
SPT_B9F6V5_ORYSJ      DQLTHVYTLIIRPDAKYSILIDNT

GROUP_5
SPT_B5M4U3_9STRA      DKDTHLYTAVIRDDNTFEVFVDQK
SPT_A9PHA6_POPTR      DKHTHVYTAILKSDNELQILVDGE
SW_CALX_PEA           DKLSHVYTAVLKDDNEVSILIDGE
SW_CALX_RAT           DKKTHLYTLILNPDNSFEILVDQS
SPT_B4R3M8_DROSI      DYLPHLYQLVVRPDNSFEIRVDHK
SPT_Q9TVF3_SCHJA      DKKTHLYTLVLRSDNSFERYIDQI
SPT_A2E253_TRIVA      DKNNHLYTLIVRPDNTFEILIDAE
SPT_A8K454_HUMAN      DRKTHLYTLVMNPDDTFEVLVDQT
SPT_Q80YU3_MOUSE      DRKTHLYTLVMNPDDTFEVLIDQK
SPT_Q7Q9V3_ANOGA      DKRPHLYQLIIRPDNTYTIRVDHK
SPT_B4JYJ8_DROGR      DKLPHLYQLVLRPDNSFEIRLDHK
SPT_D1ZZU8_TRICA      DKLPHLFTLVLHPDNTFEILIDNK
SPT_Q0KHZ9_DROME      DKLPHLYQLVVRPDNSFEIRVDHK
SPT_Q9I7S9_DROME      DKLPHLYQLVVRPDNSFEIRIDHK
SPT_B4IF87_DROSE      DKLPHLYQLVVRPDNSFEIRIDHK
SPT_Q6ZP56_HUMAN      DKKTHLYTLILNPDNSFEILVDQS
```
