# Supplementary material for: X-Ray Structure of the Human Calreticulin Globular Domain Reveals a Peptide-Binding Area and Suggests a Multi-Molecular Mechanism
Source: PLoS One. 2011 Mar 15;6(3):e17886. doi: 10.1371/journal.pone.0017886 (PMC3057994; doi:10.1371/journal.pone.0017886)
Supplement: Figure S2 — Description of the set of sequences of the CRT/CNX family. The following information about the sequence names is indicated: main protein name (when defined), source and alignment score of the whole sequence to the human globular domain sequence. The sequences have been automatically assigned to several groups using the pipealign procedure [30]. In the alignments, the sequence names directly coming from the Swissprot database have the SW prefix; the other protein sequences, translated from the gene, have the SPT prefix. (PDF) [file pone.0017886.s002.pdf]

Figure S2: Description of the set of sequences of the CRT/CNX family.
The following information about the sequence names isindicated: main protein name (when defined), source and alignment score of the whole sequence to the human globular domain sequence. The sequences have been automatically assigned to several groups using the pipealign procedure. In the alignments, the sequence names directly coming from the Swissprot database have the SW prefix; the other protein sequences, translated from the gene, have the SPT prefix.

GROUP_1

human_GB_CRT	Calreticulin	Human	user submitted sequence
A8E4J9_9ACAR	Calreticulin	Haemaphysalis qinghaiensis.	2e-84
Q64K99_9ACAR	Calreticulin	Amblyomma cooperi.	1e-83
B5X0V5_SALSA	Calreticulin	Salmo salar (Atlantic salmon).	7e-82
CALR_RABIT	Calreticulin	Oryctolagus cuniculus (Rabbit).	e-102
Q64K76_IXOPE	Calreticulin	Ixodes persulcatus (Taiga tick).	7e-81
Q7ZXY3_XENLA	Crc protein	Xenopus laevis (African clawed frog)	2e-92
Q68HD1_IXOSC	Calreticulin	Ixodes scapularis (Black-legged tick) (Deer tick).	4e-80
B5X207_SALSA	Calreticulin	Salmo salar (Atlantic salmon).	2e-87
Q64K83_9ACAR	Calreticulin	Ixodes minor.	7e-80
CALR_MACFA	Calreticulin	Macaca fascicularis (Crab eating macaque) (Cynomolgus monkey).	e-107
Q9U6S0_STRPU	Calreticulin	Strongylocentrotus purpuratus (Purple sea urchin).	1e-78
A5D7J6_BOVIN	CALR protein	Bos taurus (Bovine).	e-101
A1YB06_EISFO	Calreticulin	Eisenia foetida (Common brandling worm) (Common dung-worm).	9e-74
Q98984_RANRU	Calreticulin	Rana rugosa (Wrinkled frog).	7e-86
B3S3X6_TRIAD	Putative	Trichoplax adhaerens.	2e-73
Q6NVT6_XENTR	Calreticulin	Xenopus tropicalis (Western clawed frog) (Silurana tropicalis).	9e-90
A5LGG9_CRAGI	Calreticulin	Crassostrea gigas (Pacific oyster) (Crassostrea angulata).	4e-73
CALR_CRIGR	Calreticulin	Cricetulus griseus (Chinese hamster).	e-103
A6YIE3_PINFU	Calreticulin	Pinctada fucata (Pearl oyster).	4e-72
Q6WSQ2_RHISA	Calreticulin	Rhipicephalus sanguineus (Brown dog tick).	2e-80
Q64K91_DERAN	Calreticulin	Dermacentor andersoni (Rocky mountain wood tick).	4e-82
Q64K92_9ACAR	Calreticulin	Dermacentor albipictus.	5e-83
Q0VJ74_9BILA	Calreticulin	Heligmosomoides polygyrus.	3e-70
Q64K89_DERVA	Calreticulin	Dermacentor variabilis (American dog tick).	2e-82
O97372_DIRIM	Calreticulin	Dirofilaria immitis (Canine heartworm).	2e-69
Q9PUC1_DANRE	Calreticulin	Danio rerio (Zebrafish) (Brachydanio rerio).	6e-85
C5MRN9_9BILA	Calreticulin-like	Steinernema feltiae.	3e-68
Q26268_APLCA	Calreticulin	Aplysia californica (California sea hare).	3e-86
CALR_ONCVO	Calreticulin	Onchocerca volvulus.	7e-69
A9C3S5_DANRE	Calreticulin, like	Danio rerio (Zebrafish) (Brachydanio rerio).	7e-89
B8K275_FENCH	Calreticulin	Fenneropenaeus chinensis (Fleshy prawn) (Penaeus chinensis).	7e-73
Q8IS63_COTRU	Calreticulin	Cotesia rubecula (Cabbage white butterfly parasite).	2e-71
Q8WR36_ANOGA	Calreticulin	Anopheles gambiae (African malaria mosquito).	4e-75
Q17MI1_AEDAE	Calreticulin	Aedes aegypti (Yellowfever mosquito) (Culex aegypti).	1e-74
C1BVC1_9MAXI	Calreticulin	Lepeophtheirus salmonis (salmon louse).	9e-68
D3TM69_GLOMM	Calreticulin	Glossina morsitans morsitans (Savannah tsetse fly).	2e-74

GROUP_2

Q7Y140_ORYSJ	Os03g0832200	Oryza sativa subsp. japonica (Rice).	1e-53
A9SCB3_PHYPA	Predicted	Physcomitrella patens subsp. patens.	2e-53
CALR_BERST	Calreticulin	Berberis stolonifera (Barberry).	2e-51
C4PB36_CARPA	Calreticulin	Carica papaya (Papaya).	1e-50
CALR1_ARATH	Calreticulin-1	Arabidopsis thaliana (Mouse-ear cress).	3e-52
A9NS22_PICSI	Putative	Picea sitchensis (Sitka spruce).	5e-51
Q5MCL9_WHEAT	Calreticulin-like	Triticum aestivum (Wheat).	2e-54
O22502_BRANA	Calreticulin	Brassica napus (Rape).	2e-35
B9F6V5_ORYSJ	Putative	Oryza sativa subsp. japonica (Rice).	3e-43

Figure Supp 2 (second part)

GROUP_3			
Q4R3W0_MACFA	CALR3	Macaca fascicularis (Crab eating macaque) (Cynomolgus monkey).	2e-64
B1Q2L7_MOUSE	Calspeculin	Mus musculus (Mouse).	5e-66
CALR3_HUMAN	Calreticulin-3	Homo sapiens (Human).	6e-65
Q3TQS0_MOUSE	Calreticulin 4;	Mus musculus (Mouse).	2e-66
D3ZYM6_RAT	Calr4	Rattus norvegicus (Rat).	2e-65
C1N041_9CHLO	Predicted	Micromonas pusilla CCMP1545.	7e-53
O45034_SCHJA	Putative	Schistosoma japonicum (Blood fluke).	2e-50
C4Q5I7_SCHMA	Putative	Schistosoma mansoni (Blood fluke).	6e-52
C8XTL8_9EUKA	Calreticulin	Lotharella amoeboformis.	4e-53
D3BIF1_POLPA	Calreticulin	Polysphondylium pallidum PN500.	6e-60
CALR_EUGGR	Calreticulin	Euglena gracilis.	5e-55
C4M296_ENTHI	Putative	Entamoeba histolytica.	9e-46
B7GDH4_PHATR	Calreticulin	Phaeodactylum tricornutum CCAP 1055/1.	7e-48
Q967S4_TRYCO	Calreticulin	Trypanosoma congolense.	6e-44
A4HJP8_LEIBR	Calreticulin	Leishmania braziliensis.	7e-38
Q4Q601_LEIMA	Putative	Leishmania major.	5e-41
Q4CPZ0_TRYCR	Putative	Trypanosoma cruzi.	4e-45
C8CIJ1_9TRYP	Calreticulin	Trypanosoma carassii.	1e-44
Q9XYF8_TRYCR	Calreticulin	Trypanosoma cruzi.	2e-44
C5L9W8_9ALVE	Putative	Perkinsus marinus ATCC 50983.	4e-34
C5LE92_9ALVE	Putative	Perkinsus marinus ATCC 50983.	1e-32
C5LZP6_9ALVE	Putative	Perkinsus marinus ATCC 50983.	8e-34
D0NR25_PHYIN	Calreticulin	Phytophthora infestans T30-4.	2e-42
A8PEL1_BRUMA	Calreticulin-like	Brugia malayi (Filarial nematode worm).	4e-29
GROUP_4			
C6TCW1_SOYBN	Putative	Glycine max (Soybean).	4e-48
B6TA28_MAIZE	Calreticulin-3	Zea mays (Maize).	2e-48
C5XET1_SORBI	Sb03g042500	Sorghum bicolor (Sorghum) (Sorghum vulgare).	1e-48
B8LRK0_PICSI	Putative	Picea sitchensis (Sitka spruce).	2e-47
B4FUA8_MAIZE	Putative	Zea mays (Maize).	5e-49
C5Z0S1_SORBI	Sb09g024930	Sorghum bicolor (Sorghum) (Sorghum vulgare).	2e-46
Q8GUI1_ARATH	Calreticulin-like	Arabidopsis thaliana (Mouse-ear cress).	2e-45
B9MY68_POPTR	Predicted	Populus trichocarpa (Western balsam poplar)	7e-47
Q8LJ85_ORYSJ	Putative	Oryza sativa subsp. japonica (Rice).	2e-49
B8AZS4_ORYSI	Putative	Oryza sativa subsp. indica (Rice).	4e-46
GROUP_5			
B5M4U3_9STRA	Calcium-dep-2	Phytophthora sojae.	9e-18
A9PHA6_POPTR	Predicted	Populus trichocarpa (Western balsam poplar)	2e-11
CALX_PEA	Calnexin-like	Pisum sativum (Garden pea).	2e-13
CALX_RAT	Calnexin	Rattus norvegicus (Rat).	8e-21
B4R3M8_DROSI	GD15935	Drosophila simulans (Fruit fly).	2e-14
Q9TVF3_SCHJA	Sj66	Schistosoma japonicum (Blood fluke).	5e-18
A2E253_TRIVA	Calreticulin-like	Trichomonas vaginalis.	3e-23
A8K454_HUMAN	Calmegin-like	Homo sapiens (Human).	2e-18
Q80YU3_MOUSE	Calmegin	Mus musculus (Mouse).	4e-20
Q7Q9V3_ANOGA	AGAP005032-PB	Anopheles gambiae (African malaria mosquito).	2e-17
B4JYJ8_DROGR	GH14000	Drosophila grimshawi (Fruit fly) (Idiomyia grimshawi).	4e-15
D1ZZU8_TRICA	GLEAN_07397	Tribolium castaneum (Red flour beetle).	4e-21
Q0KHZ9_DROME	Calnexin 99A,- C	Drosophila melanogaster (Fruit fly).	7e-15
Q9I7S9_DROME	CG1924	Drosophila melanogaster (Fruit fly).	3e-12
B4IF87_DROSE	GM13395	Drosophila sechellia (Fruit fly).	7e-14
Q6ZP56_HUMAN	Calnexin-like	Homo sapiens (Human).	9e-19
