# Supplementary material for: X-Ray Structure of the Human Calreticulin Globular Domain Reveals a Peptide-Binding Area and Suggests a Multi-Molecular Mechanism
Source: PLoS One. 2011 Mar 15;6(3):e17886. doi: 10.1371/journal.pone.0017886 (PMC3057994; doi:10.1371/journal.pone.0017886)

Figure S3: Relative locations of the glucose-binding site, the peptide-binding site, cluster 2 and the main mutations affecting the *in vivo* chaperone properties.

A. Zoom on the lectin site. B. The lectin site and cluster 2 are located on two opposite sides of the beta-barrel. Mutations affecting only the lectin activity, which line the glucose-binding site (GBS) are colored magenta in A and green in B. The proposed peptide-binding site is colored orange in A, red and yellow in B. The red and orange residues in B highlight the mutations affecting the *in vivo* chaperone properties.

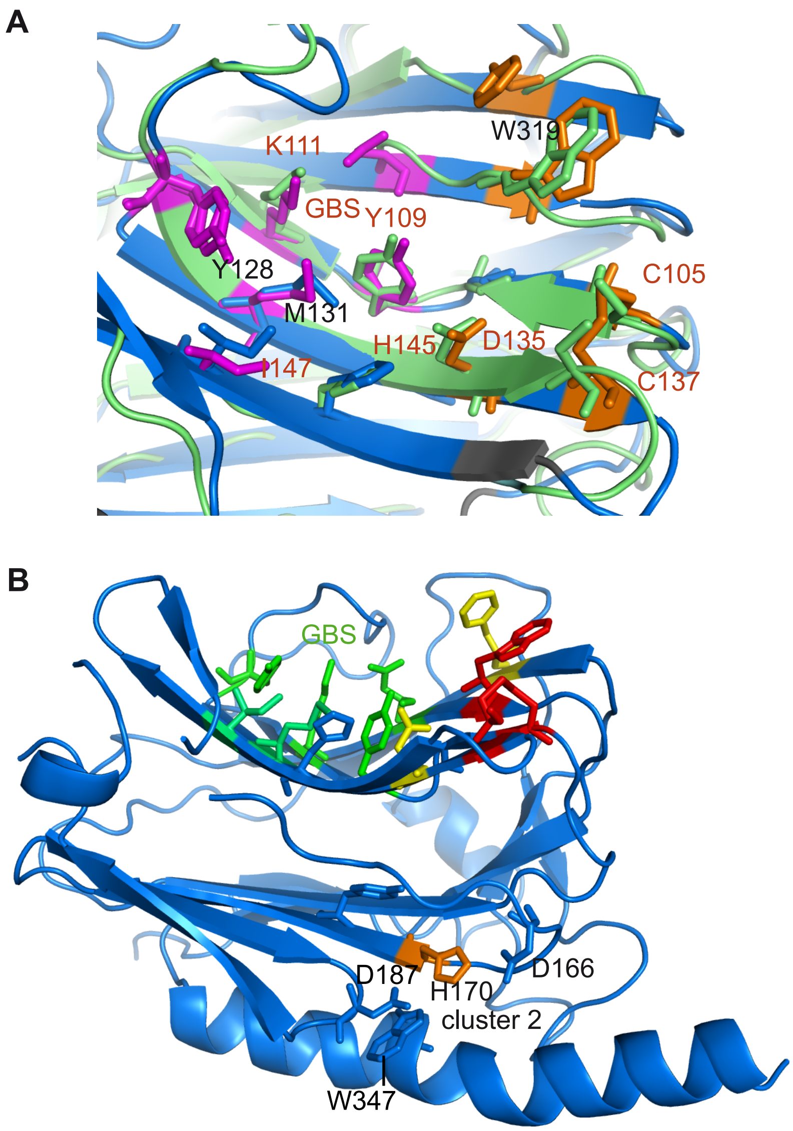

Supplement: Figure S3 — Relative locations of the glucose-binding site, the peptide-binding site, cluster 2 and the main mutations affecting the in vivo chaperone properties. A. Zoom on the lectin site. B. The lectin site and cluster 2 are located on two opposite sides of the beta-barrel. Mutations affecting only the lectin activity, which line the glucose-binding site (GBS) are colored magenta in A and green in B. The proposed peptide-binding site is colored orange in A, red and yellow in B. The red and orange residues in B highlight the mutations affecting the in vivo chaperone properties. (PDF) [file pone.0017886.s003.pdf]
