# Supplementary material for: X-Ray Structure of the Human Calreticulin Globular Domain Reveals a Peptide-Binding Area and Suggests a Multi-Molecular Mechanism
Source: PLoS One. 2011 Mar 15;6(3):e17886. doi: 10.1371/journal.pone.0017886 (PMC3057994; doi:10.1371/journal.pone.0017886)
Supplement: Figure S4 — Sequence alignments of the SQDAR 69–73 segment. This sequence alignment shows how the residues highlighted in yellow are well conserved in the CRT groups 1 and 2; less conserved in groups 3 and 4; and not conserved in the group 5. The following F74 (green) is found in most of the CRT-like sequences, except for the plant group 4. It is not present in the CNX-like group 5 sequences. Details corresponding to the sequence names are given in Figure S2. (PDF) [file pone.0017886.s004.pdf]

Figure S4: Sequence alignments of the SQDAR 69-73 segment.
This sequence alignment shows how the residues highlighted in yellow are well conserved in the CRT groups 1 and 2; less conserved in groups 3 and 4; and not conserved in the group 5. The following F74 (green) is found in most of the CRT-like sequences, except for the plant group 4. It is not present in the CNX-like group 5 sequences. Details corresponding to the sequence names are given in Figure S2.

GROUP_1

human_GB_CRT	GLQTSQDARFYAL
SPT_A8E4J9_9ACAR	GIQTSEDARFYGL
SPT_Q64K99_9ACAR	GLQTSEDARFYGI
SPT_B5X0V5_SALSA	GLQTSQDARFYAL
SW_CALR_RABIT	GLQTSQDARFYAL
SPT_Q64K76_IXOPE	GIQTSEDARFYGL
SPT_Q7ZXY3_XENLA	GLQTSQDARFYAM
SPT_Q68HD1_IXOSC	GIQTSEDARFYGL
SPT_B5X207_SALSA	GLQTSQDAHFYSS
SPT_Q64K83_9ACAR	GIQTSEDARFYGL
SW_CALR_MACFA	GLQTSQDARFYAL
SPT_Q9U6S0_STRPU	GIQTSQDAKFYGL
SPT_A5D7J6_BOVIN	GLQTSQDARFYAL
SPT_A1YB06_EISFO	GLQTTQDARFYGI
SPT_Q98984_RANRU	GLQTSQDAKFYAH
SPT_B3S3X6_TRIAD	GIQTSQDARFYAV
SPT_Q6NVT6_XENTR	GLQTSQDARFYAL
SPT_A5LGG9_CRAGI	GIQTSQDAKFYGI
SW_CALR_CRIGR	GLQTSQDARFYAL
SPT_A6YIE3_PINFU	GIQTSQDAKFYGI
SPT_Q6WSQ2_RHISA	GLQTSEDARFYGI
SPT_Q64K91_DERAN	GLQTSEDARFYGI
SPT_Q64K92_9ACAR	GLQTSEDARFYGI
SPT_Q0VJ74_9BILA	GLKTSQDAKFYSR
SPT_Q64K89_DERVA	GLQTSEDARFYGI
SPT_O97372_DIRIM	GLKTTQDAKFYSI
SPT_Q9PUC1_DANRE	GLQTSQDARFYAL
SPT_C5MRN9_9BILA	GLKTAQDARFYSI
SPT_Q26268_APLCA	IQTSQDARFYGL
SW_CALR_ONCVO	GLKTTQDAKFYSI
SPT_A9C3S5_DANRE	GLQTSQDAHFYAM
SPT_B8K275_FENCH	GIQTGQDARFYGL
SPT_Q8IS63_COTRU	GIQTTEDARFYAL
SPT_Q8WR36_ANOGA	GLQTSQDARFYAL
SPT_Q17MI1_AEDAE	GLQTSQDARFYAA
SPT_C1BVC1_9MAXI	GIQTSQDARFYAL
SPT_D3TM69_GLOMM	GLQTSQDARFYAI

GROUP_2

SPT_Q7Y140_ORYSJ	GIQTSEDYRFYAI
SPT_A9SCB3_PHYPA	GIQTSTDSRYYAI
SW_CALR_BERST	GIQTSEDYRFYAI
SPT_C4PB36_CARPA	GIQTSEDYRFYAI
SW_CALR1_ARATH	GIQTSEDYRFYAI
SPT_A9NS22_PICSI	GIQTHPDARFFAI
SPT_Q5MCL9_WHEAT	GIQTSEDYRFYAI
SPT_O22502_BRANA	RPARTTDSTPSQL
SPT_B9F6V5_ORYSJ	GIQTSEDYRFYAI

GROUP_3

SPT_Q4R3W0_MACFA	GLQTTQNGRFYAI
SPT_B1Q2L7_MOUSE	GLQTTQNSRFYAI
SW_CALR3_HUMAN	GLQTTQNGRFYAI
SPT_Q3TQS0_MOUSE	GLQTTEDAKFYAL
SPT_D3ZYM6_RAT	GLQTTEDAKFYAL
SPT_C1N041_9CHLO	GIQTSADARFYAL
SPT_O45034_SCHJA	GLKTTQDARFYGI
SPT_C4Q5I7_SCHMA	GLKTTQDARFYGI
SPT_C8XTL8_9EUKA	GIMTTQDAKFYAL
SPT_D3BIF1_POLPA	GIQTSPDARFYAI
SW_CALR_EUGGR	GIQTSQDAKFYAI
SPT_C4M296_ENTHI	GLQTSEDNKFYIA
SPT_B7GDH4_PHATR	GIQTSVDARFFGI
SPT_Q967S4_TRYCO	GLRLTEDARFYAL
SPT_A4HJP8_LEIBR	GVKLTEDSKFYAI
SPT_Q4Q601_LEIMA	GLKLMEDAKFYAV
SPT_Q4CPZ0_TRYCR	GLRLTEDARFYAL
SPT_C8CIJ1_9TRYP	GLQLTQDARFYAV
SPT_Q9XYF8_TRYCR	GLRLTEDARFYAL
SPT_C5L9W8_9ALVE	GLKTAEDHRYYAY
SPT_C5LE92_9ALVE	GLKTAEDHRYYAY
SPT_C5LZP6_9ALVE	GLKTAEDHRYYAY
SPT_D0NR25_PHYIN	AIKTSEDARFYAL
SPT_A8PEL1_BRUMA	GLTTTEDVKNYAI

GROUP_4

SPT_C6TCW1_SOYBN	GIQTSSDAKHFAI
SPT_B6TA28_MAIZE	GIQTTIDARHFAI
SPT_C5XET1_SORBI	GIQTTMDARHFAI
SPT_B8LRK0_PICSI	GIQTYPDARHYAI
SPT_B4FUA8_MAIZE	GIQTTIDARHFAI
SPT_C5Z0S1_SORBI	GIQTTGDAKHFAI
SPT_Q8GUI1_ARATH	GIQTYNDAKHYAI
SPT_B9MY68_POPTR	GIQTYNDAKHFAI
SPT_Q8LJ85_ORYSJ	GIQTTLDARHFAI
SPT_B8AZS4_ORYSI	GIQTTSDAKHFAI

GROUP_5

SPT_B5M4U3_9STRA	GLVLDKPARHYGL
SPT_A9PHA6_POPTR	GLLVSEPAKKYAI
SW_CALX_PEA	GLLVSEPARKYAI
SW_CALX_RAT	GLVLMSRAKHHAI
SPT_B4R3M8_DROSI	GLVLKSKAKHAAI
SPT_Q9TVF3_SCHJA	ALVLKSKAKHHAV
SPT_A2E253_TRIVA	LFKMKAASKYYGA
SPT_A8K454_HUMAN	GLVLKSRAKHHAI
SPT_Q80YU3_MOUSE	GLVLKSKAKHHAI
SPT_Q7Q9V3_ANOGA	GLVLKSKAKHAAI
SPT_B4JYJ8_DROGR	GLVLKSKAKHAAI
SPT_D1ZZU8_TRICA	GLVLKSKAKHAAI
SPT_Q0KHZ9_DROME	GLVLKSKAKHAAI
SPT_Q9I7S9_DROME	GLVLKSKAKHAAI
SPT_B4IF87_DROSE	GLVLKSKAKHAAI
SPT_Q6ZP56_HUMAN	GLVLMSRAKHHAI
